# Vital Signal Detection Using Multi-Radar for Reductions in Body Movement Effects

**DOI:** 10.3390/s21217398

**Published:** 2021-11-07

**Authors:** Ah-Jung Jang, In-Seong Lee, Jong-Ryul Yang

**Affiliations:** Department of Electronic Engineering, Yeungnam University, Gyeongsan 38541, Korea; dkwjd289@yu.ac.kr (A.-J.J.); dldlstjd0322@yu.ac.kr (I.-S.L.)

**Keywords:** body movement cancelation, continuous wave, Doppler radar, multiple radars, vital signal detection, heartbeat, respiration

## Abstract

Vital signal detection using multiple radars is proposed to reduce the signal degradation from a subject’s body movement. The phase variation in the transceiving signals of continuous-wave radar due to respiration and heartbeat is generated by the body surface movement of the organs monitored in the line-of-sight (LOS) of the radar. The body movement signals obtained by two adjacent radars can be assumed to be the same over a certain distance. However, the vital signals are different in each radar, and each radar has a different LOS because of the asymmetric movement of lungs and heart. The proposed method uses two adjacent radars with different LOS to obtain correlated signals that reinforce the difference in the asymmetrical movement of the organs. The correlated signals can improve the signal-to-noise ratio in vital signal detection because of a reduction in the body movement effect. Two radars at different frequencies in the 5.8 GHz band are implemented to reduce direct signal coupling. Measurement results using the radars arranged at angles of 30°, 45°, and 60° showed that the proposed method can detect the vital signals with a mean accuracy of 97.8% for the subject moving at a maximum velocity of 53.4 mm/s.

## 1. Introduction

The extraction of respiration and heartbeat signals from the variations in transceiving signal characteristics is a promising remote vital signal detection technology because it can escape the physical restraint of a contact sensor and be used in various applications [1,2,3]. A continuous wave (CW) Doppler radar, which monitors the Doppler frequency change in the CW signal caused by the thoracic movement from respiration and the heart’s periodic movement, can acquire remote vital signals using a simple hardware configuration [4,5].

The effect of a subject’s body movement must be removed for vital signal detection technology using a CW Doppler radar sensor to be commercialized for industrial and medical applications. A human body movement normally involves a larger displacement than movement caused by respiration (with a displacement of 4–12 mm) and heartbeat (with a displacement of 0.2–0.5 mm) on the body surface [6]. When a human body moves, a signal saturation may occur in a sensitive receiver for heartbeat detection because of its limited dynamic range, making it impossible to detect any signals [7]. Even when the radar has a sufficiently wide dynamic range, the frequency components of body movement can occupy a similar band to the frequencies resulting from respiration and heartbeat. As these components act as noise, they can deteriorate the signal-to-noise ratio (SNR) in vital signal detection or make the detection of vital signals impossible [7].

Previous studies on mitigating performance degradation in vital signal detection due to body movement can be divided into techniques for improving radar hardware configuration and signal-processing techniques [8,9,10,11,12,13,14,15,16,17,18,19]. Previous studies on radar hardware configuration separate the body movement and vital signals by measuring the directivity of body movement. Some studies use a plurality of radars to remove Doppler shifts due to body movement by arranging them at positions facing each other around the subject and comparing the phase change in the baseband signals obtained from each radar [8,9,10,11,12]. However, these studies have a limitation in that it is difficult to consider the same movement in each radar because of the interference between radars and a change in the polarity of the received signal. Previous studies have shown that body movement can be canceled by fusion techniques using additional sensors, but they have a limitation in terms of their increased system complexity and implementation cost [13,14,15,16]. A signal-processing technique for minimizing the effect of body movement on radars is based on the compensation of the dominant baseband signal characteristics generated by body movement [17,18,19]. They have a limitation in that compensating for the effect of body movement because the cancelation performance can depend on the windowing size and time period of polynomial fitting. Although previous studies on removing the effect of body movement in vital signal detection using radars have been conducted in various directions, a technique for removing the effect of body movement has not been sufficiently explored.

In this study, the method of placing two independent radar sensors at the front for a certain angle of line-of-sight (LOS) is proposed to effectively compensate for the body movement characteristics and sensitively detect only vital signals based on the asymmetrical movement of internal organs. In the proposed radar configuration, two radars with different LOS are arranged in the same direction within a shorter distance than the wavelength of the operating frequency. The two radars use different operating frequencies to minimize direct coupling. It is assumed that the vital signals obtained by the two radars are different because of the asymmetric movement of organs, but the signal from body movement is approximately the same for each radar. The proposed configuration can improve the SNR of vital signal detection by removing the baseband signals from body movement. Section 2 describes the displacement difference due to the asymmetric movement of the heart and lungs, along with the proposed configuration and operating principle using multiple radars. The digital signal processing and hardware configuration to improve the SNR using a correlation between the two baseband signals of the radars are presented in Section 3. The measurement results and analyses are discussed in Section 4. Section 5 presents the conclusions of this study.

## 2. Proposed Configuration Using Multi-Radars

### 2.1. Physiological Movement of the Heart and Lungs

**Figure 1:**
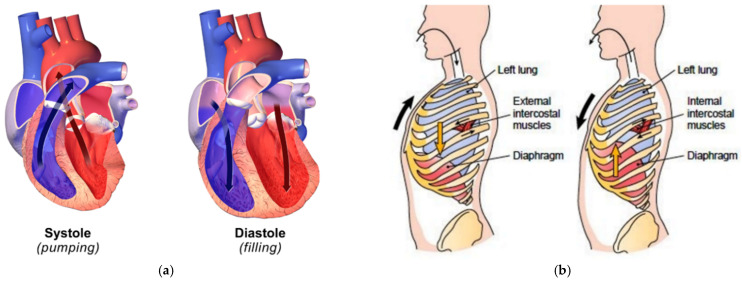
Asymmetrical movements of human organs: (**a**) pumping and filling of blood in the heart; (**b**) expansion and contraction in the lungs [25,26].

The heart and lungs inside a human body move in asymmetric directions with repeated contraction and expansion, as shown in Figure 1. The human heart in Figure 1a, which is divided into four parts (left and right atria and left and right ventricles) generates different displacements of the chest wall due to its different volumes and pressures [20,21,22]. In addition to the non-uniform characteristics of the human tissue layer consisting of various organs, muscles, and bones, the inherent directional movement of heart muscles caused by its various parts is asymmetrically monitored on the surface of the human body. As shown in Figure 1b, the lung movement during respiration is accompanied by the movement of the surrounding intercostal muscles, diaphragm, and the lung itself, resulting in a larger asymmetrical movement. The movement due to respiration, accompanied by the movement of the ribcage, is also generated anisotropically because the body volume depends on the contraction and expansion of the lungs [23,24]. The asymmetric movements of the human heart and lungs imply that a radar sensor to detect vital signals from varying surface displacements can be positioned along a specific direction to increase its detection sensitivity.

**Figure 2:**
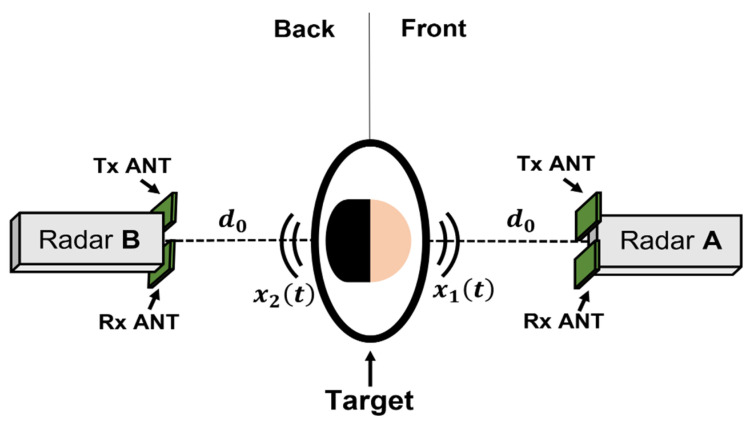
Preceding experiment showing that the respiration and heartbeat signals obtained from the radar can be different depending on the direction of the line-of-sight to the subject.

**Figure 3:**
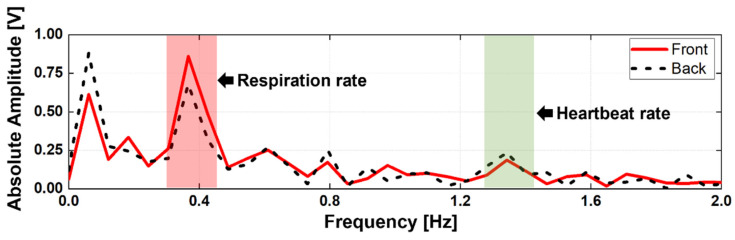
Frequency spectrum of the vital signals simultaneously measured from the two radars placed in front of and behind the subject.

The asymmetric movement of the heart and lungs on the surface of the human body was experimentally verified using two 5.8-GHz CW Doppler radars, as shown in Figure 2, to monitor respiration and heartbeat signals from a periodic displacement. The configuration of the CW Doppler radar module is described in Section 3. Two radars operating at the same frequency are placed between the subject in a line at a distance of 0.8 m to ensure their LOS is between the subject’s front and rear. It was assumed that the vital signals obtained from each radar have dominant characteristics caused by the position of the human body. Unlike previous studies, in which radars were also placed on the left and right sides of the subject, in this study, there were no additional radars on either side to exclude the effect of minute movements of the subject′s arms [9]. Figure 3 shows the vital signals that were simultaneously measured by the radars. Both the measured data displayed respiration and heartbeat signals at the same frequency, but the signal powers were measured differently between the two datasets, even though all components and conditions in the radars were identical. The respiration measured from the front radar (located at the front of the subject) had a higher intensity than that from the rear radar (located on the back of the subject). However, the heartbeat measured from the rear radar had a higher intensity than that from the front radar, even though the noise signal near DC was higher in the rear radar. The measurement results in Figure 3 show that the respiration and heartbeat signals measured by the radars, which detect the vital signal from the displacement of the human body surface, have an asymmetrical movement, as shown in several previous studies [8,9,10]. It is unreasonable to insist that the rear radar is more advantageous for heartbeat detection than the front radar based on the results in Figure 3. The SNR for heartbeat detection could decrease even though the amplitude of the heartbeat signal increased in the rear radar because it could increase the harmonic components of respiration and the noise near DC by increasing the nonlinearity of the received signal. The SNR could improve by using a signal correlation between the front and rear radars, because the body movement could be canceled by a displacement compensation using the quadrature signals of the configuration shown in Figure 3; however, the compensation performance is limited when the received signal from the rear radar is too small to detect the vital signals of the subject. The vital signals from the rear radar generally have lower power due to the small displacements based on the asymmetric movements of human organs when compared to the front radar, and their attenuation is more affected in the rear radar by the clothing conditions of the subject and the distance between the subject and the radar.

### 2.2. Proposed Configuration for Vital Signal Detection Using Multiple Radars

**Figure 4:**
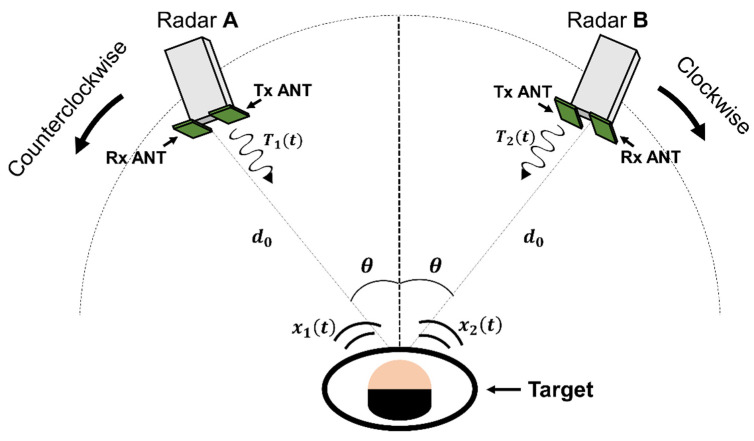
Proposed radar configuration using multiple radars based on the asymmetrical movement of human organs.

A configuration using multiple radars, as shown in Figure 4, is proposed to increase the SNR of vital signal detection while considering the asymmetrical movements of human organs. The radar modules in the 5.8 GHz frequency band, which operate independently and consist of transmitting and receiving front-end and baseband circuits, are separately located at the same angle (θ) to the left and right and at the same distance (*d_0_*) from the subject. The operating frequencies of the two radars are set to be different within the frequency band to reduce the degradation from a direct signal coupling between them and are arranged at an angle of 30° or more for a sufficient separation distance to reduce the increase in noise due to the blocker signal. When the operating frequencies of the two radars are different in the frequency band, the transmitted signals *T*_1_(*t*) and T_2_(*t*) from the two radars can be expressed as follows:

(1) $$T_{k}\left(t\right)=A_{\mathrm{Tk}}\cdot \cos\left[2{\pi f}_{k}t+\theta_{k}\left(t\right)\right],\;k=1.2,$$

where *k* is an index to discriminate the radar module, *f_k_* is the operating frequency of each radar, *A_Tk_* is the amplitude of the transmitted signals, and ∆*θ_k_*(*t*) is the phase noise generated from the signal source at the operating frequency. The vital signals generated by the asymmetrical movements of human organs are differently monitored in the two radars because of their different LOSs, and the received signals *R_k_*(*t*) in each radar can be expressed, except for the non-ideal characteristics such as the multipath and signal coupling, as follows:

(2) $$R_{k}\left(t\right)=A_{\mathrm{Rk}}\cdot \cos\left[2{\pi f}_{k}t-\frac{4{\pi d}_{0}}{\lambda_{k}}-\frac{4{\pi x}_{k}\left(t\right)}{\lambda_{k}}\pm \frac{4\pi b\left(t\right)}{\lambda_{k}}+\theta_{k}\left(t-\frac{2d_{0}}{c}\right)\right],\;k=1.2$$

where *A_Rk_* denotes the amplitudes of the received signals, *c* represents the propagation velocity of light in air, *λ_k_* denotes the wavelength of the operating frequency, *x_k_*(*t*) denotes the displacement of the vital signals, and *b*(*t*) denotes the displacement caused by human body movement. Assuming that the human body moves only in the forward and backward directions, the displacement *b*(*t*) because of this movement can be equally expressed in both radars. The ± sign is used to indicate the human body movement direction, and the + and – signs, respectively, indicate movements approaching and moving away from the radar. Owing to the asymmetric movements of the heart and lungs and the different radar-operating frequencies, *x_k_*(*t*) can be expressed by distinguishing the amplitudes, phases, and frequencies as follows:

(3) $$x_{k}\left(t\right)=x_{rk}\left(t\right)+x_{hk}\left(t\right)=m_{rk}\cos\left(\omega_{r}t+\varphi_{rk}\right)+m_{hk}\cos\left(\omega_{h}t+\varphi_{hk}\right)$$

where *x_rk_*(*t*) and *x_hk_*(*t*) are the displacements from respiration and heartbeat, respectively; *m_rk_* and *m_hk_* denote the magnitudes of respiration and heartbeat, respectively; *w_r_* and *w_h_* denote the angular frequencies of respiration and heartbeat, respectively; and *φ_rk_* and *φ_hk_* denote the phases of respiration and heartbeat, respectively. Although the radars are located at the same distance from the subject, the magnitudes *m_rk_* and *m_hk_* and the phases *φ_rk_* and *φ_hk_* are differently indicated because of the asymmetrical movement of the organs. The angular frequencies *w_r_* and *w_h_* can be assumed to be a single-frequency component because respiration and heartbeat signals at the surface of the human body are dominated by changes in the volume of the chest cavity and the left ventricle’s movement, respectively [20,24]. After a down-conversion with a quadrature mixer and filtering with the low-pass filters, the baseband signals in the in-phase (I) and quadrature (Q) channels can be obtained as:

(4) $$I_{k}\left(t\right)=A_{Ik}\cdot \cos\left[\frac{4{\pi d}_{0}}{\lambda_{k}}+\frac{4{\pi x}_{k}\left(t\right)}{\lambda_{k}}\mp \frac{4\pi b\left(t\right)}{\lambda_{k}}+\cdot \theta_{k}\left(t\right)\right]+{DC}_{Ik},\;k=1.2$$

(5) $$Q_{k}\left(t\right)=A_{Qk}\cdot \sin\left[\frac{4{\pi d}_{0}}{\lambda_{k}}+\frac{4{\pi x}_{k}\left(t\right)}{\lambda_{k}}\mp \frac{4\pi b\left(t\right)}{\lambda_{k}}+\cdot \theta_{k}\left(t\right)\right]+{DC}_{Qk},\;k=1.2$$

where *A_Ik_* and *A_Qk_* are the amplitudes in I/Q channels, ∆*θ_k_*(*t*) is the residual phase noise, which is neglected in short-range applications because of the range correlation effect, and *DC_IK_* and *DC_QK_* are the DC offset voltages in I/Q channels, which are generated by stationary clutters in the experimental environment and direct coupling between the transmitting and receiving signals [27]. The signal processing may require demodulation to extract the vital signal *x*(*t*) in the trigonometric functions in Equations (4) and (5). A mathematical demodulation technique such as arcsine or arctangent demodulation is not suitable for the proposed configuration with multiple radar modules because DC offset voltages caused by the presence of the subject and surrounding clutter are difficult to remove [27,28]. The circle fitting method, which can extract the displacement through the circle trajectory, shown as a graph in the I/Q plot, can demodulate the dominant displacement of baseband signals, even in an environment with a DC offset [29]. However, when *b*(*t*) caused by the movement of the human body is dominantly shown in the circle trajectory, the circle fitting method has limits to vital signal detection using the proposed radar configuration because *x*(*t*) may be lost in the demodulated signals [8]. The complex signal demodulation (CSD) used in the proposed configuration is useful for detecting small displacements from vital signals in an environment with a DC offset. The complex signal *S_k_*(*t*) can be expressed as follows:

(6) $$S_{k}\left(t\right)=I_{k}^{\prime }\left(t\right)+j\cdot Q_{k}^{\prime }\left(t\right)=A_{k}\exp\left[j\left(\frac{4\pi d_{0}}{\lambda_{k}}+\frac{4\pi x_{k}\left(t\right)}{\lambda_{k}}\mp \frac{4\pi b\left(t\right)}{\lambda_{k}}\right)\right],\;\;k=1.2$$

where *I_k_′*(*t*) and *Q_k_′*(*t*) are baseband signals after compensating for the I/Q imbalance, and *A_k_* is the amplitude of the complex signal. The DC offset is negligible in the I/Q imbalance compensation and the CSD method because it is significantly reduced in *S_k_*(*t*) by the amplitude of the baseband signals.

A signal processing technique is proposed to improve the SNR in vital signal detection using the correlation between two radar signals. By normalizing *S_k_*(*t*) and extracting only the phase using a natural logarithm, the phase of the baseband signal obtained in the conventional CSD method can be expressed as follows:

(7) $$P_{k}\left(t\right)=\frac{4\pi d_{0}}{\lambda_{k}}+\frac{4\pi x_{k}\left(t\right)}{\lambda_{k}}\mp \frac{4\pi b\left(t\right)}{\lambda_{k}}.\;\;\;\;\;\;\;\;\;k=1.2$$

The phase difference *P_D_*(*t*) between two operating frequencies obtained from each radar can be expressed as

(8) $$P_{D}\left(t\right)=P_{1}\left(t\right)-P_{2}\left(t\right)=4\pi \left(d_{0}\mp b\left(t\right)\right)\cdot \;\left(\frac{1}{\lambda_{1}}-\frac{1}{\lambda_{2}}\right)+4\pi \left(\frac{x_{1}\left(t\right)}{\lambda_{1}}-\frac{x_{2}\left(t\right)}{\lambda_{2}}\right)$$

As shown in Equation (8), the effect of *b*(*t*) in the proposed configuration is not entirely diminished because of the different operating frequencies, but it is smaller than that in the single CW radar configuration. The amplitude of the periodic vital signal is as prominent as the asymmetrical movement of organs due to the non-identical amplitudes of the vital signals from each radar. When an identical operating frequency is used in the two radars, the effect of the human body movement can be removed, as the second term in Equation (8) will remain due to the asymmetric movement, but the first term will cancel out [8,9,10]. However, the displacement of the movement in Equation (8) is difficult to remove if the wavelength difference between the two radars is large, and it has a significant effect on the noise level by increasing the harmonic components due to the vital signals and the signal caused by human body movement. The mathematical expression of the signal using Equation (8) can be expressed as

(9) $$C\left(t\right)=\exp\left(jP_{D}\left(t\right)\right)=\exp\left[j4\pi \left(d_{0}\mp b\left(t\right)\right)\cdot \;\left(\frac{1}{\lambda_{1}}-\frac{1}{\lambda_{2}}\right)+j4\pi \left(\frac{x_{1}\left(t\right)}{\lambda_{1}}-\frac{x_{2}\left(t\right)}{\lambda_{2}}\right)\right]$$

The conventional signal processing method obtains the respiration and heartbeat signals by searching the signal amplitude in the frequency band corresponding to the respiration and heartbeat using the fast Fourier Transform (FFT) of Equation (9). Respiratory and heartbeat signals can be accurately obtained for each vital signal by comparison with the frequency detected by the reference sensor. In the proposed signal processing, the vital signals are extracted from the difference in the spectrum obtained by each FFT of the demodulated signal by the CSD. The mathematical expression of the signal processed by the proposed method can be expressed as a subtraction of the normalized complex signals *S_k_′*(*t*) shown in Equation (6) as follows:

(10) $$F\left(t\right)=S_{1}^{\prime }\left(t\right)-S_{2}^{\prime }\left(t\right)=\exp\left[\frac{j4\pi }{\lambda_{1}}\left(d_{0}+x_{1}\left(t\right)\mp b\left(t\right)\right)\right]-\exp\left[\frac{j4\pi }{\lambda_{2}}\left(d_{0}+x_{2}\left(t\right)\mp b\left(t\right)\right)\right].\;\;$$

The first term in the exponential function represents the DC signals caused by the distance between the radar and the subject; the DC signal level from the difference between two frequencies is reduced in the FFT results compared with that from a single radar. The effect of *b*(*t*) in the proposed configuration is not entirely diminished because of the different operating frequencies, but *b*(*t*) in Equation (10) can be expressed in the same form as Equation (9) after applying a complex FFT and can be reduced in the output signal *F*(*t*) by a subtractive operation. However, *x*(*t*) in Equation (10) cannot be reduced at the output because *x*(*t*) in each radar is not the same in magnitude and phase, as shown in Equation (3). As the phases of the vital signals independently obtained from the two radars are different owing to the asymmetric movement of human organs, *x*(*t*) may be integrated during the sampling period and increase beyond the signal level obtained from a single radar.

**Figure 5:**
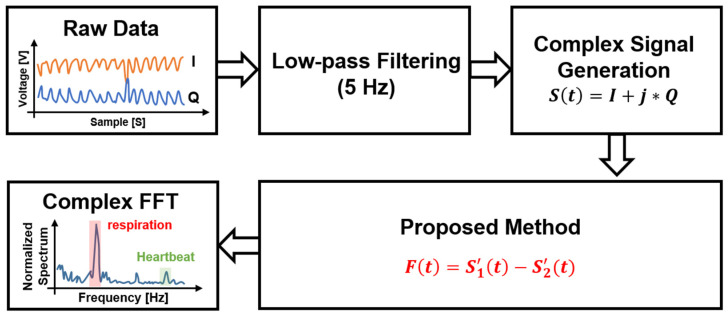
Digital signal processing using the proposed correlation technique for reducing the effect of human body movement.

**Figure 6:**
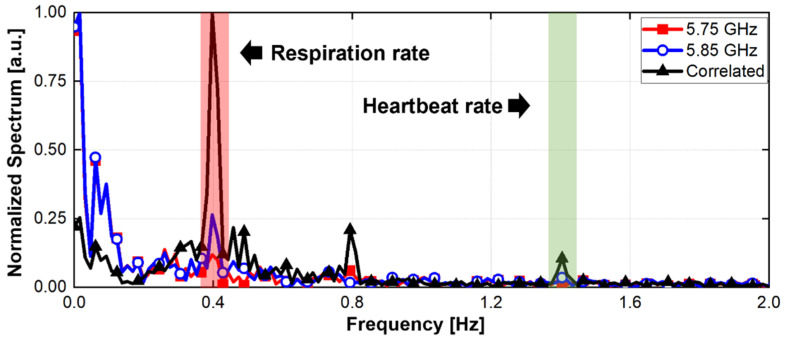
Normalized spectrum of baseband signals in the simulation.

**Figure 7:**
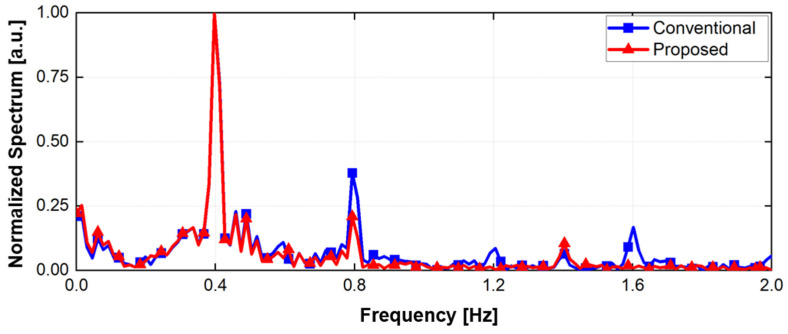
Simulated spectrum of the conventional and proposed signal processing methods.

**Figure 8:**
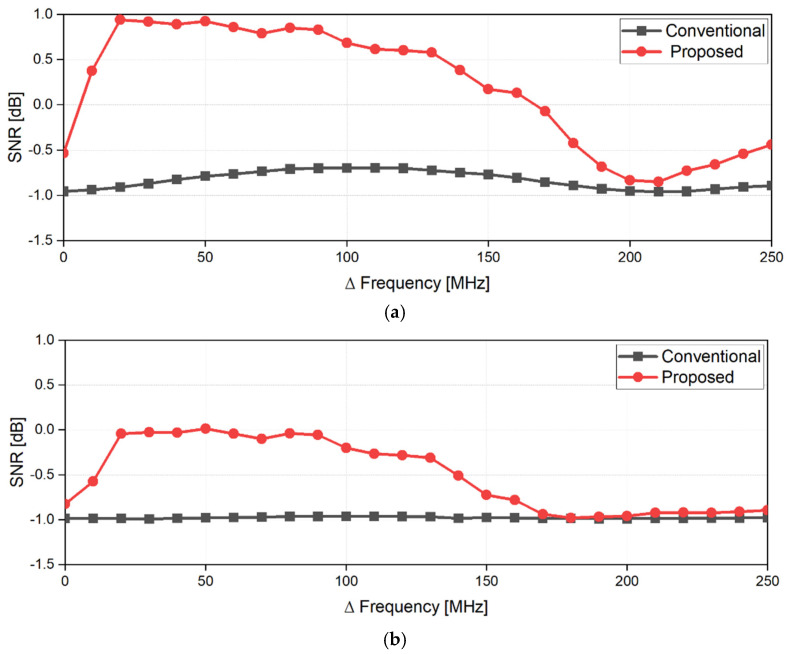
Signal-to-noise ratios of the conventional and proposed signal processing methods depending on the difference in the operating frequency of the two radars: the signal is the magnitude of the vital signs and the noise is the magnitude of the signal caused by the human body movement: (**a**) for respiration; (**b**) for heartbeat.

Figure 5 shows the digital signal processing in the proposed radar configuration. A simulation to verify the proposed configuration and signal processing was performed using MATLAB. It was assumed that two radars individually operating at 5.75 GHz and 5.85 GHz are located at 0.5 m from the subject. In the simulation, the magnitudes of the vital signals were set to 8 mV_PP_ at radar A and 7 mV_PP_ at radar B for respiration and 1 mV_PP_ at radar A and 0.4 mV_PP_ at radar B for heartbeat, considering the measurement data from the previous experiments [4,5,18]. The phase differences of the vital signals were set to π between radar A and B. The overall data acquisition time and sampling frequency in the simulated experiment were set to 40 s and 1 kHz, respectively. The simulation assumed that the signal caused by the human body is located at 0.003 Hz with the magnitude of 100 mV_PP_. Figure 6 shows the normalized spectrum of the baseband signals using Equations (6) and (10) following the complex FFT. In the simulated spectrum of the single-frequency radar, the signal caused by body movement has a lower frequency than the vital signals because of its low velocity. The simulation results show that the SNR of the vital signals can be reduced by body movements with a large displacement. The simulated spectrum processed by the proposed signal processing method shows a significant reduction in the effect of body movement because of a correlation between the baseband signals of the two radars. A respiration frequency of 0.4 Hz and heartbeat frequency of 1.4 Hz, which are the same values as in the simulation condition, were effectively recovered by increasing the SNR of the vital signal detection because of the proposed signal processing. Figure 7 shows the simulated spectra of both the conventional method and the proposed method in the radar configuration. Compared to the conventional method, which increases the harmonics using a nonlinear function, the modified method can suppress the harmonic generation in the spectrum. The same frequencies of vital signals in both spectra indicate that the proposed method does not distort the demodulated signals when compared to the conventional method. Figure 8 shows the signal processing gain of the conventional and proposed signal processing methods depending on the difference in the operating frequency of the two radars. The SNR in Figure 8 was calculated from the simulation results by using the signal spectrum magnitude of the vital signals and the noise spectrum magnitude of the human movement signal. Simulation results show that the proposed signal processing method can achieve higher SNR in both respiration and heart rate compared to the conventional method. In particular, the SNR of the proposed method improved 1.3 dB for respiration signals and 0.7 dB for heartbeat signals at a frequency difference of 100 MHz in the proposed configuration. The SNRs of the conventional and proposed methods do not show a significant difference above the frequency difference of 170 MHz. These results show that the proposed method can be effective for vital signal detection in the 5.8 GHz ISM band with a maximum frequency bandwidth of 150 MHz.

## 3. Measurement Environment

**Figure 9:**
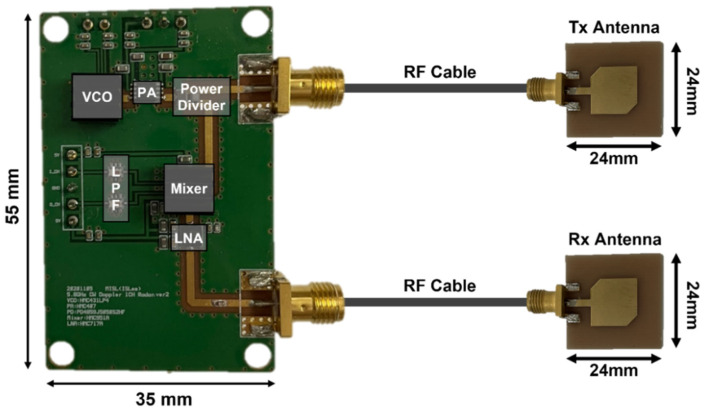
Implemented single-channel radar module in the 5.8 GHz ISM band.

**Figure 10:**
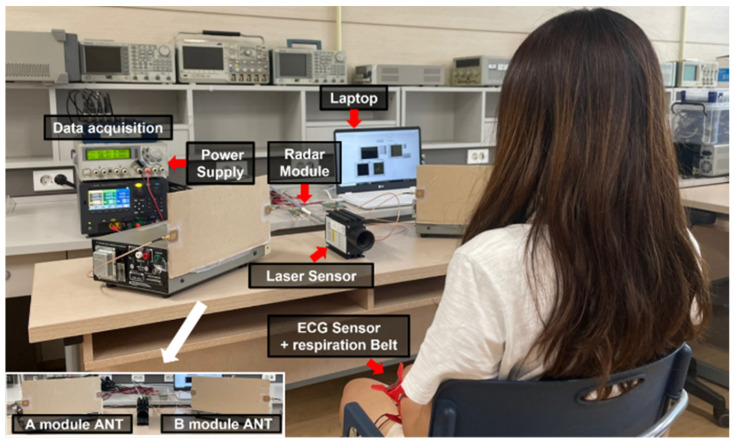
Experiment environment of the proposed radar configuration for vital signal detection for canceling the effect of body movement.

Two signal channel radar modules were implemented for the proposed radar configuration as shown in Figure 9 [17]. The operating frequencies of the two radars in the 5.8 GHz ISM band are individually determined with the control voltage of a voltage-controlled oscillator (VCO). In the experiment, the frequencies were set to 5.75 GHz and 5.85 GHz, with a frequency gap of 100 MHz. The transmitted powers at each radar module were measured to be 7.8 dBm at 5.75 GHz and 9.3 dBm at 5.85 GHz, respectively. The desired LOS, as shown in Figure 10, was set to the position and angle of the patch antenna with an antenna gain of 4.4 dBi, which is connected to the radar module through low-loss RF cables. The quadrature signals of the module were simultaneously obtained using a data acquisition board (NI USB-6366, National Instruments, Austin, TX, USA) with a sampling rate of 1 k samples per second in each channel of the two radars. A three-electrode ECG sensor (EKG-BTA, Vernier Software & Technology, Beaverton, OR, USA) and respiration belt (GDX-RB, Vernier Software & Technology, Beaverton, OR, USA) were used as reference sensors to compare the accuracy of vital sign detection using the proposed radar configuration. The reference data for displacement and velocity of the human body movement were measured using a laser sensor (ILR1182-30, Micro-Epsilon, Ortenburg, Germany) with a resolution of 0.1 mm and sampling rate of 50 samples per second.

The two CW radars with different operating frequencies were positioned at a specific angle in the radar configuration, as shown in Figure 4 and Figure 10. The angles in the experiment were set to 30°, 45°, and 60°, and the distance between the subject and each radar was fixed at 0.5 m. The body movement was controlled in the experiment for the given conditions, a motionless state and a random back-and-forth moving state for approximately 5 s. The movement of the subject’s arms was restricted because the movement may affect the experimental results. The velocity obtained from the reference laser sensor was calibrated to the velocity at the radar module considering the different LOSs and the angles.

## 4. Results and Discussion

**Figure 11:**
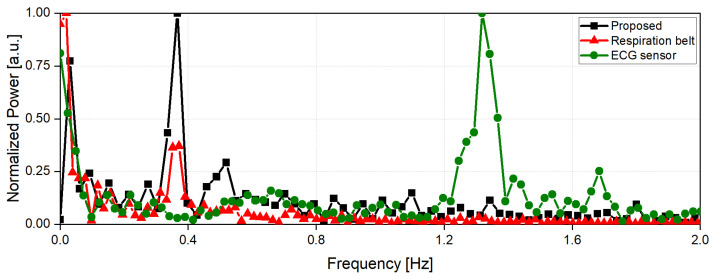
Frequency spectra of the vital signals measured by using the reference sensors (a respiration belt and an ECG sensor) and the proposed radar configuration at the LOS angle of 30°.

The radar experimental frequency spectrum of the respiration and heartbeat signals were simultaneously compared with the signals of the reference respiration belt and an ECG sensor. The experiment was configured so that the subject′s respiration harmonics did not overlap with the heartbeat signal to prevent obscuring the detection of the heartbeat signal. Figure 11 shows the frequency spectra of the vital signals obtained from the proposed radar configuration with a LOS angle of 30° and the reference signals obtained from the respiration belt and the ECG sensor. The peak frequency of the respiration in the proposed radar was 0.36 Hz while the reference frequency measured by the respiration belt was 0.37 Hz. The peak frequency of the heartbeat signal in the radar was 1.34 Hz while the reference ECG sensor measured 1.32 Hz. The frequency difference between the respiration harmonics and the heartbeat was 0.1 Hz or higher.

**Figure 12:**
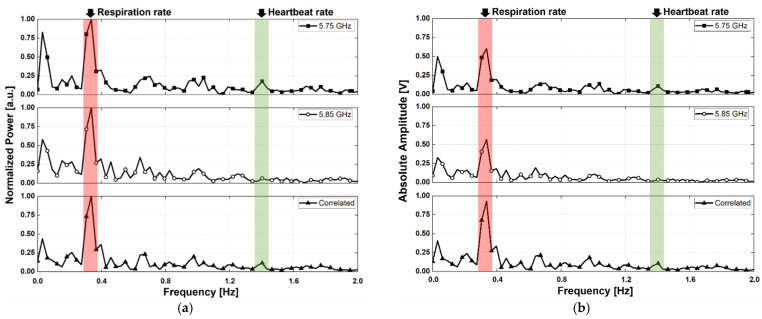

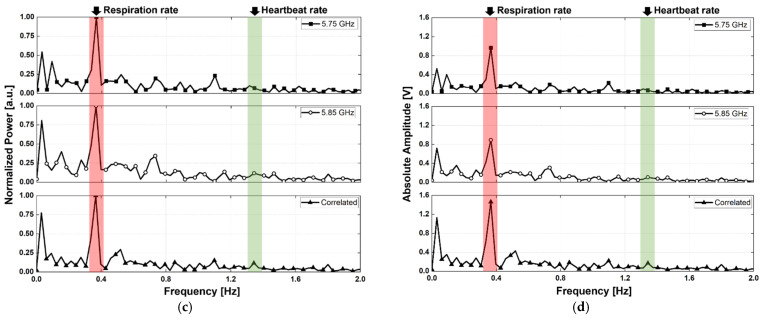
Measurement results using the proposed radar configuration in the experiment with a measurement angle of 30°: (**a**) normalized spectrum obtained in the motionless condition; (**b**) spectrum displayed with the absolute amplitudes of signals in the motionless condition; (**c**) normalized spectrum obtained in the presence of human body movement; and (**d**) spectrum displayed with the absolute amplitudes of the signals in the presence of human body movement.

**Figure 13:**
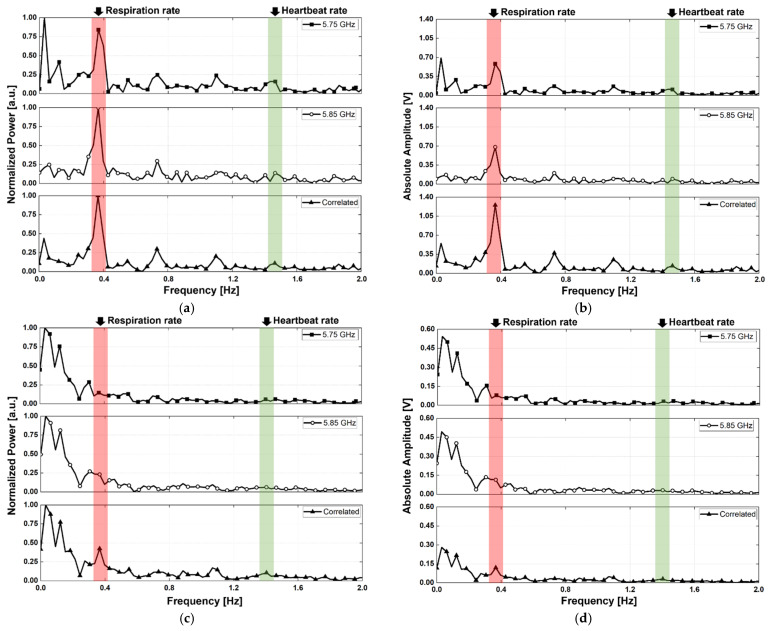
Measurement results using the proposed radar configuration in the experiment with a measurement angle of 45°: (**a**) normalized spectrum obtained in the motionless condition; (**b**) spectrum displayed with the absolute amplitudes of signals in the motionless condition; (**c**) normalized spectrum obtained in the presence of human body movement; (**d**) spectrum displayed with the absolute amplitudes of the signals in the presence of human body movement.

**Figure 14:**
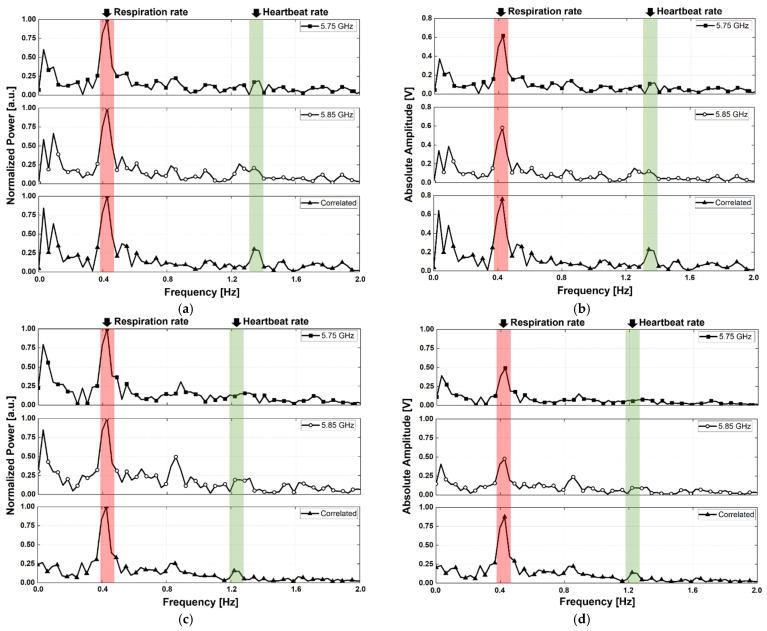
Measurement results using the proposed radar configuration in the experiment with a measurement angle of 60°: (**a**) normalized spectrum obtained in the motionless condition; (**b**) spectrum displayed with the absolute amplitudes of signals in the motionless condition; (**c**) normalized spectrum obtained in the presence of human body movement; (**d**) spectrum displayed with the absolute amplitudes of the signals in the presence of human body movement.

The vital signals obtained in the proposed configuration using two radars were presented as a spectrum depending on the LOS, angle, and presence of body movement. The measurement results are of two types: a normalized spectrum and a spectrum expressed with the absolute amplitude, for demonstrating the SNR improvement of the vital signal detection by the proposed radar configuration and signal processing. Figure 12, Figure 13 and Figure 14 show the spectra measured in the experimental environment configured with a measurement angles of 30°, 45°, and 60°, respectively. In the case of motion, the subject in the experiment moved arbitrarily in the forward and backward directions, and the maximum velocity in the measurement was presented with a laser-based reference sensor because it is difficult to control the velocity and displacement of these movements. Owing to the angle set by the LOS of the subject and the radar, the body movement of the subject is presented in the direction of the LOS on the radar. The velocity of the subject’s body movement was measured to be a maximum of 17.2 mm/s at 30°, 12 mm/s at 45°, and 26.7 mm/s at 60° using the reference sensor at the front of the subject, and the calculated velocity considering the angle was 19.9 mm/s at 30°, 17.0 mm/s at 45°, and 53.4 mm/s at 60°, respectively. The subject′s movement was limited in the experiment because a sufficient space between the radars and the subject was not secured, and the maximum displacement because of the movement was 80 mm at 30°, 80 mm at 45°, and 30 mm at 60°, as measured by the reference sensor.

Respirations were detected from the measured signal peaks, regardless of the presence or absence of motion in the LOS angles of 30° and 60°, as shown in Figure 12a,c, Figure 13a and Figure 14a,c. However, in the radar arrangement at an angle of 45°, the peak of the respiration signal was detected only in the correlated signal by the proposed signal processing, as shown in Figure 13c. Despite its small velocity when compared to other angles, the subject′s movement at 45° in Figure 13 had a significant effect on vital signal detection because of the SNR degradation caused by an increase in the noise. This shows that the displacement magnitude is more important than velocity of human body movement because the subject at an angle of 45° moved his body with a high displacement and a low velocity. For the subject’s motionless condition, the heartbeat signals were measured at all angles by a correlation between the two radars using the proposed method, as shown in Figure 12a, Figure 13a and Figure 14a; however, the signal in the single radar was measured only from radar A, which was located close to the subject′s heart. Assuming that the transmitter output power and receiver sensitivity of the two radars do not have a significant difference, it can be seen that a larger heartbeat signal was received by radar A because of the asymmetry of the heart rate and position between the two radars. The heartbeat signals in an environment with the subject′s motion were not obtained from each radar, as shown in Figure 12c, Figure 13c and Figure 14c. However, the signal correlated using the proposed signal processing displayed the heartbeat signals at all angles. The performance improvement by the proposed configuration and processing can be explained by the increase in the SNR because of a decrease in the noise reduction near DC. Figure 12d, Figure 13d and Figure 14d, displayed as the absolute amplitudes of the signals, show that the signals near DC caused by the motion of the subject are significantly reduced by the proposed signal processing. The spectra at angles of 30° and 45° (Figure 12b and Figure 13b) in the motionless condition show that the noise level near DC was slightly reduced by the proposed method. However, there was no significant reduction in the noise level of the spectrum at an angle of 60°, as shown in Figure 14b. The residual noise near DC in the motionless state of the subject shows that the proposed configuration and processing can only improve the performance for common noise in both radars. This shows the limitation of the proposed configuration and processing: it does not show a performance improvement for noise generated by the asymmetric clutter and multipath problem.

The SNR of the vital signal detection in the experiment can be expressed as the ratio of the sum of the respiration and heartbeat signals to the sum of all signals except the vital signals below 2 Hz. The SNR improvement of the vital signal detection by the proposed signal processing method for the moving human body condition was measured to be 5.6 dB for respiration and 3.3 dB for heartbeat at an angle of 30°, 5.7 dB for respiration and 4.2 dB for heartbeat at an angle of 45°, and 3.7 dB for respiration and 3.0 dB for heartbeat at an angle of 60°, respectively. The detection accuracy of the vital signal calculated using the measured peaks in Figure 12, Figure 13 and Figure 14 was 96.8% for respiration and 98.2% for heartbeat at 30°, 96% for respiration and 99.2% for heartbeat at 45°, and 98.4% for respiration and 96% for heartbeat at 60°, respectively. Compared to previous studies on vital signal detection using the CW radars, the detection accuracy of the proposed radar configuration was lower for respiration but higher for heartbeat. In the measurement results, the detection accuracy of respiration was reduced by increasing the noise level because of the movement of the human body appearing at a lower frequency range than the respiration. The detection accuracy of heartbeat was not affected by the noise level from the motion because of the far frequency range between the noise and the heartbeat signals and increased because of the SNR improvement from the proposed signal processing. Table 1 summarizes the comparison of vital signal detection using radar technology to reduce the effect of human body movement.

**Figure 15:**
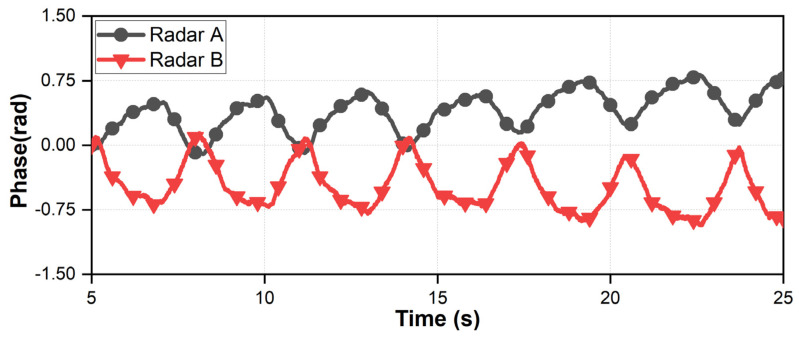
Phase waveform of the vital signals simultaneously measured in each radar.

The measurement results in the motion of the subject in Figure 12d, Figure 13d and Figure 14d show that the proposed configuration and processing increase the absolute amplitudes of the vital signals and decrease the motion-induced noise. The amplitude and phase of the vital signals obtained from the two radars should be different because of the asymmetrical movement of the human organs to simultaneously realize a decrease in noise and an increase in the vital signals. The different amplitudes and phases between the vital signals obtained from the two radars show that the asymmetric movement of human organs affects vital signal detection using the radar. In particular, the phases of the vital signals *x*_1_(*t*) and *x*_2_(*t*) acquired simultaneously by two radars should not be the same to increase the amplitude of vital signals by the proposed signal processing method explained in Section 2. Figure 15 shows the phase waveforms of vital signals acquired simultaneously and independently from the two radars. The waveforms of the simultaneously sampled data show that the phases of the vital signals have a difference of 180° in the proposed configuration. This is caused by the asymmetrical characteristics of vital signals in the radar because of the presented phase differences in the measurement results, regardless of the LOS angles.

The proposed radar configuration was demonstrated by strictly controlling the variables and factors that can affect the vital signal detection. Therefore, it has limitations for extension to general applications. However, the configuration limitations on the location and the arrangement between the radars and the subject can be solved with a modified radar operation that can detect the distance difference between each radar and the subject, such as FSK or FMCW radars. This study shows that the radar can detect vital signals based on the asymmetric movements of the internal organs using the proposed configuration and signal processing techniques.

## 5. Conclusions

A configuration and signal processing method using two radars operating at different frequencies are proposed for detecting vital signals during the subject’s body movement. Based on the asymmetrical organ movements caused by the vital signals monitored in the CW Doppler radar, the proposed radar configuration includes two radars spaced apart in front of the subject with different LOSs at the same distance. The operating frequencies of two radars were individually set to 5.75 GHz and 5.85 GHz in the 5.8 GHz ISM for reducing a direct coupling between them. A signal processing method was proposed for effectively extracting correlated vital signals from the complex baseband signals received from the two radars. The proposed signal processing had the same demodulation performance as the conventional method without using a nonlinear function, which increases the harmonics. A SNR improvement was observed in vital signal detection during human body movement, and the stable accuracy was enhanced for asymmetrical organ movements in the proposed method. The proposed configuration based on the asymmetrical movement of organs was verified by placing two radars at a distance of 0.5 m from the subject for different LOSs at angles of 30°, 45°, and 60° from the center of the subject. For the motionless condition, the respiration and heartbeat were obtained from the signals detected by the radar located closer to the heart on the left side of the subject, and the signals from the two radars were correlated using the proposed method. In the presence of body movement with a maximum velocity of 53 mm/s, respiration and heartbeat could be detected only from the correlated signals obtained using the proposed method. The noise reduction in the low-frequency range by the proposed method shows that it can reduce the effect of human movement. The improvement in SNR and the detection accuracy of both respiration and heartbeat detection by the proposed configuration and method were measured to be more than 3 dB and 96% at all three angles, respectively.

## Figures and Tables

**Table 1:** Radar technology for vital signal detection in the presence of human body movement.

Ref.	Techniques	Maximum Body Movement [mm]	Maximum Body Velocity [mm/s]	Detection Accuracy [%]	
				Respiration	Heartbeat
[8]	CSD method using two antennas around the subject	Not mentioned	4	Not mentioned	Not mentioned
[30]	SIL ^1^ radar using two antennas	200	<7.7	Not mentioned	96.5
[31]	Polynomial fitting algorithm	150	≈ 0	Not mentioned	Not mentioned
[32]	Adaptive noise cancelation algorithm	155	47.6	97.9	99.1
This work	Correlation method using multiple radars	80	53.4	97.8 ^2^	97.9 ^2^

^1^ Single Self-Injection-Locked Radar. ^2^ Average data from the measurement results at all angles.

## Data Availability

The data presented in this study are available on request from the corresponding author. The data are not publicly available due to the privacy of the subjects.
